# Clinicopathological Implications of Mismatch Repair Status in Endometrioid Endometrial Cancer in Duhok City

**DOI:** 10.7759/cureus.56861

**Published:** 2024-03-25

**Authors:** Ghorbat S Ali, Rafil T Yaqo, Mahdi A Abdullah

**Affiliations:** 1 Department of Biology, College of Science, University of Duhok, Duhok, IRQ; 2 Department of Pathology, College of Medicine, University of Duhok, Duhok, IRQ; 3 Department of Pathology & Microbiology, College of Veterinary, University of Duhok, Duhok, IRQ

**Keywords:** mismatch repair, immunohistochemistry, endometroid cancer, protein, histology, mmr

## Abstract

Background: DNA mismatch repair (MMR) is a specialized system that corrects errors in DNA replication, namely, base substitution mismatches and minor insertion-deletion mismatches. The deficient mismatch repair (d-MMR) protein plays a vital role in predicting the prognosis of endometrioid carcinoma. The study aimed to determine the prevalence of MMR errors in endometrial cancer (EC) and their correlation with clinicopathological features.

Methods: We examined the immunohistochemistry presence of four MMR proteins in 50 samples of EC tissues that were preserved in formalin and embedded in paraffin. The proteins identified were MutL homolog 1 (MLH1), post-meiotic segregation increased 2 (PMS2), MutS homolog 2 (MSH2), and MutS homolog 6 (MSH6). The study examined several clinicopathological characteristics and conducted MMR phenotyping.

Results: The findings revealed that among the 50 cases of EC, 40% of patients had grade I disease and 78% had stage I malignancy. Furthermore, among the 50 individuals evaluated, 56% exhibited competence in MMR, whereas 44% displayed loss in nuclear expression of MMR. The rate of MLH1 and PMS2 protein loss was recorded as the greatest, at 18%, while the loss of MSH2 and MSH6 was documented at 6%. Within the same range, the majority of patients with d-MMR were above the age of 50 years.

Conclusion: The majority of the recruited EC patients in this study showed advanced age and a high percentage of d-MMR status.

## Introduction

Endometrial cancer (EC) is a heterogeneous group of tumors that originate from the endometrial glandular epithelium. These tumors can be classified into several histological categories, each of which varies in the morphological characteristics they exhibit [1]. According to the Global Cancer Statistics 2020 report, EC was the sixth most common kind of cancer found in females all over the world [2].

Around 30 years ago, Bokhman was the first person to identify the two distinct types of EC [3]. The majority of cases (70-80%) are type I carcinomas, which are defined by low-grade endometrioid histology, hormone receptor positivity, early stage at diagnosis, and a favorable prognosis. On the other hand, type II carcinomas are classified by non-endometrioid histology, advanced stage at diagnosis, increased risk of metastases, and a poor prognosis [4]. It has been determined that there are a number of subtypes of endometrioid carcinoma, some of which include secretory, villoglandular, and squamous differentiation forms [5].

A molecular categorization of EC is used in place of the conventional hormone dualism of the EC to achieve better results. One of the new assays verifies the condition of DNA mismatch repair (MMR). The MMR protein system is produced by four necessary genes. These genes are MutS homolog 2 (MSH2), MutL homolog 1 (MLH1), MutS homolog 6 (MSH6), and post-meiotic segregation increased 2 (PMS2). The MMR proteins are responsible for correcting and protecting against DNA mutations. When the mechanism is defective, there is a significant rise in the number of single nucleotide variants as well as minor insertion and deletion changes. This characteristic is referred to as a deficient mismatch repair (d-MMR) system or high microsatellite instability (MSI) [6,7]. MMR deficiency can be tested using immunohistochemistry (IHC) for MMR protein expression, polymerase chain reaction assessment of MSI, and next-generation sequencing [8].

On the other hand, in contrast to the MSI approach, the IHC method is technically less complicated, less expensive, and more easily accessible. Furthermore, it provides information regarding the MMR gene that is affected [9]. The grade of the tumor, the histological subtype, the degree of myometrial invasion, the involvement of the cervical region, the size of the tumor, lymphovascular space invasion (LVSI), and the status of the lymph nodes are the most important prognostic indicators for patients with EC [10]. There were a number of studies that observed the relationship between d-MMR and factors such as advanced age, larger tumors, higher grade, higher stage disease, deeper LVSI, and invasive myometrium [11,12]. In contrast, the other investigations revealed that there were no alterations in the stage, grade, or LVSI [13].

It is of the utmost significance to conduct research on new molecular markers that are capable of predicting responses to conservative treatment. An investigation into the role of MMR proteins as a promising predictive biomarker for conservative treatment has only been conducted in a limited number of studies involving small populations [14]. There is a limited amount of data that recorded the MMR expression status in the EC in our regions. Therefore, the current study aims to achieve an IHC study using a four-antibody panel for detecting the percentage of d-MMR in EC patients in Duhok province and to assess its correlation with certain clinicopathologic characteristics.

## Materials and methods

Sample collection

This study was approved by the Scientific Committee of Duhok University/College of Science (reference number: 1732) and the Ethical Committee of the General Directorate of Health in Duhok under reference number 24102021-10-37. All patients who had a full hysterectomy between February 2016 and June 2023 were included in the study that analyzed the histology of surgical specimens and selected them as cases of EC.

Clinicopathological data

The clinicopathological information relevant to these individuals was retrieved from their medical records. This information included the age at which the tumors were discovered, the grade, the stage, myometrial invasion, and LVSI. A total of 50 samples that were embedded in paraffin and had been formalin-fixed were collected from Vajeen Specialist Laboratory and Duhok Central Health Laboratory, both of which are classified as pathology laboratories. The sections of the tumor that offered the most precise representation were chosen for further examination.

Histopathology examination

The tissue blocks were sectioned and stained with standard hematoxylin and eosin. The thickness of the sections was three microns. The histological investigation was carried out in a manner that was consistent with the recommendations that were provided by the World Health Organization [15].

Tissue microarray

According to the methodology that was described in the past, tissue microarray (TMA) is a method that may be utilized to analyze protein expression in tissue sections at a high throughput [16]. Through the use of Beech equipment, small cores of formalin-fixed, paraffin-embedded (FFPE) tissue were extracted from a large number of "donor" paraffin blocks. These cores were organized in a new "recipient" TMA. Twelve samples representing a variety of donor specimens were included in each TMA. After cutting tissue sections with a thickness of 3 micrometers, each of these sections was stained simultaneously with IHC for a specific marker.

Immunohistochemistry

Tissue microarray sections of FFPE block specimens were used to detect MMR proteins. Tissue microarray slides were incubated at 60°C for two hours. Ten-minute deparaffinization in xylene was performed twice on tissue sections. The tissue slices were then rinsed with distilled water after being rehydrated with graded alcohols for five minutes each. The next stage of antigen retrieval was performed in a Dako PT link tank loaded with a pH 9 Tris-EDTA (ethylenediaminetetraacetic acid) buffer at 97°C for 20 minutes. After washing, the slides were put in FLEX, a peroxidase enzyme inhibitor. This solution contained 0.3% hydrogen peroxidase to suppress endogenous peroxidase.

Dako-Agilent (Glostrup, Denmark) made ready-to-use antibodies from DAKO FLEX for all four biomarkers. A 20-minute incubation was used for MLH1 and MSH6, while a 30-minute incubation was used for MSH2 and PMS2. Slides were then cleaned with Tris-buffered saline (TBS). After that, a dextran polymer-horseradish peroxidase dye was added to the mixture and left for 20 minutes (MLH1), 30 minutes (MSH2), 20 minutes (MSH6), and 30 minutes (PMS2). After 10 minutes in FLEX DAB chromogen plus sub-chromogen solution, the slides were washed twice in TBS buffer for five minutes each and rinsed in distilled water. The stained tissue slices were counterstained with hematoxylin. Positive internal control included lymphocytes and normal stromal cells. All proteins were expressed in proficient MMR (p-MMR) patients, although at least one of four proteins failed to express in MMR-deficient cases.

Analysis based on statistics

An analysis was performed to determine the frequency and proportion of qualitative variables. Through the utilization of the chi-squared test and Fisher's exact test, the association that existed between the expression status of the MMR protein and the clinicopathological characteristics was successfully ascertained. At a significant level of p ≤ 0.05, the two-sided significance was established.

## Results

Several clinicopathologic indicators were gathered and analyzed for each of the 50 patients who participated in the study.

Histological findings

**Figure 1:**
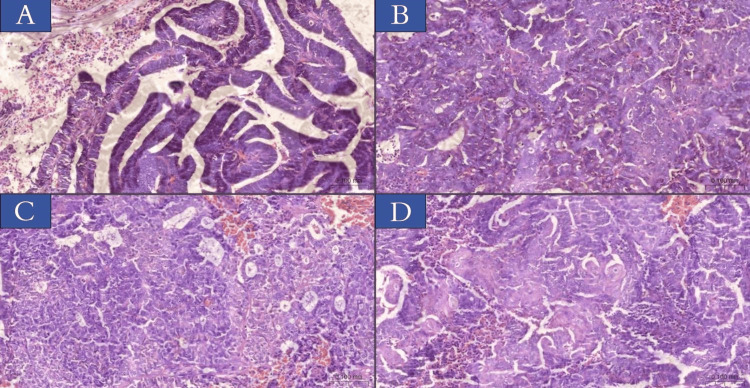
Microscopical features of endometrioid carcinoma were obtained from patient samples included in this study. Images showing morphology and grade. (A) FIGO I with villoglandular growth pattern. (B) FIGO II with acinar growth pattern. (C) FIGO III with solid growth pattern. (D) FIGO II with squamous metaplasia. Hematoxylin & eosin, x20 (slide viewer from 3dhistech.com). FIGO: International Federation of Gynecology and Obstetrics.

According to the findings of the histological analysis (Table 1), every case was identified as EC. Following a histological investigation, villoglandular patterns were found in 14 out of 50 cases (28%). On the other hand, squamous metaplasia was found in nine out of 50 cases (18%). Additionally, leiomyomas were found in six of the 50 EC patients (12%). There were around 17 patients (34%) who presented with atypical hyperplasia out of a total of 50 patients. Figure 1 illustrates this information in its respective formats.

**Table 1:** Histological study of endometrioid cancer of 50 cases.

Histological pattern	Types	No.	%
Endometrioid cancer	Villoglandular	14	28%
	Squamous metaplasia	9	18%
Endometrioid cancer with leiomyoma		6	12%
Endometrioid cancer with adenomyosis		4	8%
Endometrioid cancer with endometrioid hyperplasia atypia		17	34%

Clinicopathological features

Age at Diagnosis

In the course of the research, it was revealed that the ages of the patients ranged from 30 to 70 years, with the average age being 58 years. Of the 50 cases, eight patients (16%) were younger than 50 years old, while the remaining 42 individuals (84%) were older than 50 years old.

Tumor Location

We found eight cases (16%) of EC confined to the fundus and seven cases (14%) located within the corpus, while the majority (18 cases, 36%) were found to occupy both corpus and fundus; on the other hand, the tumor location was not mentioned in the pathological report of 17 cases (34%), which we categorized as an unspecified location within the uterus.

Grade and Stage

According to the microscopic histologic findings, out of the 50 cases that were investigated, 20 cases (40%) displayed grade I, 17 cases (34%) exhibited grade II, and 13 cases (26%) were recorded as grade III. According to the archive data of the enrolled patients, the majority of cases, which accounted for 78% of the total, were defined as being in stage I, followed by 20% of patients classified as being in stage III, while only 2% were in stage IV, and none was recorded for stage II disease.

Myometrial Invasion and Angiolymphatic Invasion

Out of 50 cases, 33 (66%) showed tumor infiltration in less than 50% equivalent to half of the myometrial thickness. In 17 cases (34%), more than 50% of myometrium was involved in the cancer. Tumor cells demonstrated lymphovascular infiltration in three (6%) of the instances. Furthermore, one of the cases (2%) exhibited a distant metastasis. Table 2 provides a summary of these findings when taken together.

**Table 2:** Clinicopathological features of studied cases. FIGO: International Federation of Gynecology and Obstetrics.

Clinicopathological parameters	Variable	No.	%	Year
Age	Mean age			58
	Less than 50	8	16%	
	Equal or more than 50	42	84%	
Location	Fundus	8	16%	
	corpus	7	14%	
	Fundus and corpus	18	36%	
	Unspecified	17	34%	
Grade	I	20	40%	
	II	17	34%	
	III	13	26%	
Myometrium invasion	>50%	33	66%	
	≤50%	17	34%	
FIGO stage	I	39	78%	
	III	10	20%	
	IV	1	2%	
Angiolymphatic invasion		3	6%	
Distant metastasis		1	2%	

Immunohistochemical analysis findings

**Figure 2:**
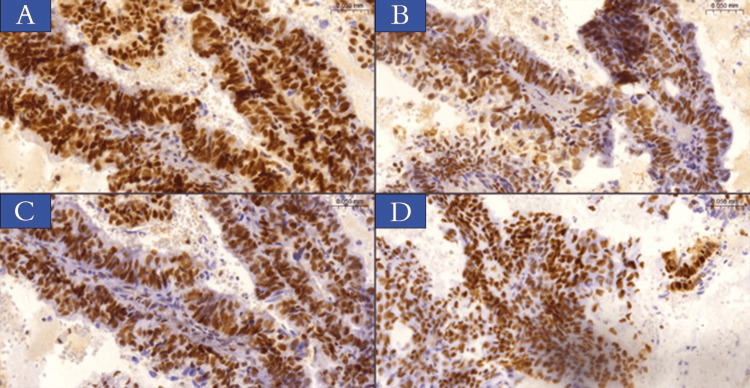
IHC staining of MMR proteins in endometrioid carcinoma sections obtained from patients involved in the current study. (A) MLH1, (B) PMS2, (C) MSH2, (D) MSH6 - normal nuclear protein expression. The magnification power is x20 (slide viewer from 3dhistech.com). IHC: immunohistochemistry; MMR: mismatch repair; MLH1: MutL homolog 1; PMS2: post-meiotic segregation increased 2; MSH2: MutS homolog 2; MSH6: MutS homolog 6.

**Figure 3:**
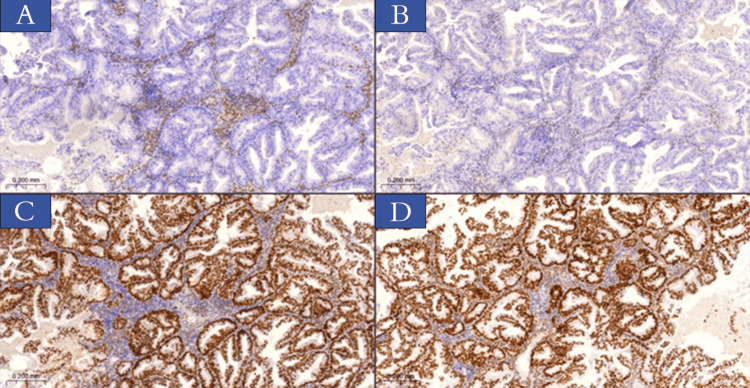
IHC staining of MMR proteins in Endometrioid cancer sections obtained from patients involved in the current study. Internal control includes stromal cells and lymphocytes expressing nuclear protein for all biomarkers. (A) Loss of nuclear expression of MLH1. (B) Loss of nuclear expression PMS2 protein. (C) Preserved nuclear expression of MSH2. (D) Preserved nuclear expression of MSH6 protein, x10 (3dhistech). IHC: immunohistochemistry; MMR: mismatch repair; MLH1: MutL homolog 1; PMS2: post-meiotic segregation increased 2; MSH2: MutS homolog 2; MSH6: MutS homolog 6.

**Figure 4:**
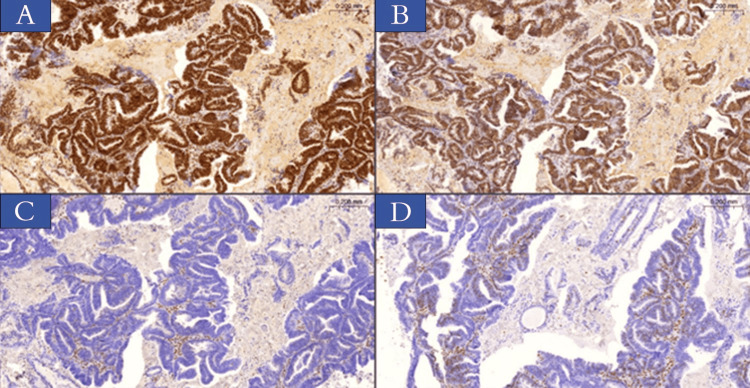
IHC staining of MMR proteins in endometrioid cancer sections obtained from patients involved in the current study. Internal control includes stromal cells and lymphocytes expressing nuclear protein for all biomarkers. (A) Preserved nuclear expression of MLH1 protein. (B) Preserved nuclear expression of PMS2 protein. (C) Loss of nuclear expression of MSH2 protein. (D) Loss of nuclear expression of MSH6 protein, x10 (3dhistech). IHC: immunohistochemistry; MMR: mismatch repair; MLH1: MutL homolog 1; PMS2: post-meiotic segregation increased 2; MSH2: MutS homolog 2; MSH6: MutS homolog 6.

Immunohistochemical analysis of the four protein markers revealed that for p-MMR for EC cases, exceedance of MMR protein status was observed in 28 cases (56%), whereas d-MMR protein status was present in 22 patients (44%). The following protein expressions were observed: nine samples (18%) exhibited paired losses of MLH1 and PMS2, while three cases (6%) revealed paired losses of MSH2 and MSH6. Moreover, three cases (6%) demonstrated three markers loss (MLH1, PMS2, and MSH6 or MSH2), and lastly, two samples (4%) showed the deficiency of four markers (MLH1, PMS2, MSH2, and MSH6). Table 3 and Figures 2-4 illustrate the results of marker expression with percentage rates.

**Table 3:** MMR expression in endometrioid endometrial cancer. MMR: mismatch repair; MLH1: MutL homolog 1; PMS2: post-meiotic segregation increased 2; MSH2: MutS homolog 2; MSH6: MutS homolog 6.

Markers expression	No.	%
MMR proficient	28	56%
MMR deficient	22	44%
MLH1 loss only	0	0
PMS2 loss only	4	8%
MSH2 loss only	1	2%
MSH6 loss only	0	0
MLH1 and PMS2 loss	9	18%
MSH2 and MSH6 loss	3	6%
Loss of 3 markers (MLH1, PMS2, and MSH6)	2	4%
Loss of 3 markers (MLH1, PMS2, and MSH2)	1	2%
Loss of 4 markers (MLH1, PMS2, MSH2, and MSH6)	2	4%

Correlation of MMR status with clinicopathological characteristics

The results of our analysis regarding the subset of endometrioid cancer patients with d-MMR and p-MMR, which correlate with clinical parameters, are presented in Table 4. No correlation between the presence or absence of MMR deficiency and mean age at the diagnosis (p = 0.32), tumor grade (p = 0.43), myometrium invasion (p = 0.14), or stage (p = 0.63) was found to be statistically significant.

**Table 4:** Correlation of MMR status with clinicopathological characteristics under variation. MMR: mismatch repair; d-MMR: deficient mismatch repair; p-MMR: proficient mismatch repair.

Parameters		Variable	d-MMR			p-MMR			p-value
			No.	%	Years	No.	%	Years	
Age	At the diagnosis	Mean + SD	22		59.5 + 1.8	28		56.9 + 2	0.32
	Category	≥50	20	90.1%		22	78.6%		0.24
		<50	2	9.1%		6	21.4%		
Grade		I	11	50%		9	32.1%		0.43
		II	6	27.3%		11	39.2%		
		III	5	22.7%		8	28.6%		
Myometrium invasion		≤50%	17	77.3%		16	57.1%		0.14
		>50%	5	22.7%		12	42.9%		
Stage		I	17	77.3%		22	78.6%		0.63
		II	0			0	0		
		III	5	22.7%		5	17.9%		
		IV	0	0%		1	3.6%		

Individuals diagnosed with d-MMR deficiency had an average age of 59.5 years, whereas those with proficient p-MMR had an average age of 56.9 years. Over the age of 50 constituted the majority of patients diagnosed with EC, comprising 90.1% of the total. However, a higher propensity for grade I was noted in d-MMR tumors (n = 11/22, 50%) as opposed to p-MMR tumors (n = 9/28, 32.1%). Grade II d-MMR tumors were identified in six out of 22 cases (27.3%), whereas grade II p-MMR tumors were identified in 11 out of 28 cases (39.2%). Furthermore, a tendency toward a higher percentage of grade III tumors was noted in d-MMR cases (n = 5/22, 22.7%) in comparison to p-MMR cases (n = 8/28, 28.6%). d-MMR tumors exhibited a considerably higher incidence of deep myometrial invasion (n = 5/22, 22.7%) in comparison to p-MMR tumors (n = 12/28 instances, 42.9%).

In the d-MMR and p-MMR groups, the incidence of stage I EC was comparable at 77.3% and 78.6%, respectively. There was a non-significant difference in MMR protein expression among patients with stage III cases. Of the patients, 17.9% with p-MMR were present in stage III, while 22.7% of patients with d-MMR were present in stage III. Only 3.6% of p-MMR patients had a stage IV disease.

## Discussion

According to Hashmi et al. [17], EC represents a significant factor in the overall sickness and mortality rates in South Asian countries with females. MMR proteins can be detected in a person who has been diagnosed with endometrioid cancer by the use of IHC screening, which is advocated for by a number of countries throughout the world. Although the majority of research on a significant number of d-MMR in EC has been carried out on Western populations, a few findings have also arisen from Asia [18-20].

IHC was utilized to ascertain the prevalence of MMR protein deficit. It was determined that the tumor samples did not contain any MMR protein expression (MLH1, PMS2, MSH2, and MSH6), which is evidence of MMR dysfunction [21]. In previous research, MMR-protein loss was detected in 16% to 45% of patients with EC using IHC [22,23]. According to this investigation, d-MMR protein was detected in 44% of the EC samples. This finding was greater than the rate discovered by Puangsricharoen et al. in 2020 when they discovered that it was 35.9% [24]. It was also comparable to a study conducted in Thailand [25].

We investigated the relationship between MMR status and clinic-pathologic characteristics. Our research showed that d-MMR endometrioid carcinoma is more common in older patients, which is in line with what other studies [26,27] have found. Other studies have found that younger patients with endometrioid carcinoma are more likely to have an MMR deficiency [28,29].

In this study, no statistically significant difference was seen between the p-MMR and d-MMR despite prognostic variables such as age, grade, stage, angiolymphatic invasion, and distant metastasis. These results were consistent with Küçük et al. and Puangsricharoen et al. [24,30]. On the other hand, research has hypothesized that cases of d-MMR are connected to older age, more advanced stages, and higher grades [31]. In their study, Kim et al. identified conflicting findings regarding the association of MMR defects with uterine cancer. They reported that d-MMR was linked to a higher histologic grade (II-III) but a lower stage (I-II) [32]. We found that our patient with MMR-deficient adenocarcinoma of endometrium was associated with low FIGO (International Federation of Gynecology and Obstetrics) grade and early-stage presentation, and these findings are comparable to previous studies on EC [30,33].

Among our patients, the prevalence of d-MMR was found to be substantially higher than average, probably due to the low sample size and missing data like lymph node metastasis status in patient records due to malpractice, thus a larger study might be warranted to give an accurate understanding of the prognostic and treatment implications of MMR-deficient EC and to assist in the formation of definitive management guidelines, additional investigations, including trials with planned designs, are required.

Limitations

Limitations of this study include a small sample size, which makes it challenging to obtain the results and may impact the statistical power. The other limitations include the data collection method, delay reports, missing family history of related malignancy, and data accuracy.

## Conclusions

Our result established that the patients with d-MMR had clinicopathologic characteristics that were connected with endometrioid histological patterns, younger age, low grade, positive angiolymphatic invasion, low distant metastasis rate, and low stage. Further prospective larger-scale studies that use molecular analysis of MMR gene defect should be done to validate these results and evaluate their prognostic and predictive value.
